# Apathy in Alzheimer's disease

**DOI:** 10.1016/j.cobeha.2017.12.007

**Published:** 2018-08

**Authors:** Lisa Nobis, Masud Husain

**Affiliations:** Nuffield Dept Clinical Neurosciences & Dept Experimental Psychology, University of Oxford, United Kingdom

## Abstract

Apathy is the most common neuropsychiatric symptom in patients with Alzheimer's disease (AD). The presence of apathy has been related to greater caregiver distress, decreased quality of life, and increased morbidity. Here we review the most recent studies on this neuropsychiatric syndrome, focusing on prevalence, impact on quality of life, behavioural and neuroimaging studies, and treatment options. The results of some investigations on the behavioural and neuroanatomical profile of apathy in AD point to a role of frontostriatal circuits, specifically involving the anterior cingulate cortex. However, small and heterogeneous samples, lack of control for disease severity, and non-specific apathy scales complicate interpretation of results. Future studies might benefit from studying multiple dimensions of apathy within conceptual frameworks which allow for a deconstruction of underlying mechanisms.

## Highlights

• Apathy is the commonest neuropsychiatric symptom in Alzheimer's disease (AD).
• Neuroimaging findings implicate frontostriatal circuits involving the anterior cingulate cortex.
• But the results are variable and interpretation is difficult for several reasons.
• Future studies might benefit from dissection of underlying behavioural mechanisms.

**Current Opinion in Behavioral Sciences** 2018, **22**:7–13

This review comes from a themed issue on **Apathy and motivation**

Edited by **Christopher Pryce** and **Masud Husain**

For a complete overview see the Issue and the Editorial

Available online 27th December 2017

**https://doi.org/10.1016/j.cobeha.2017.12.007**

2352-1546/© 2017 The Authors. Published by Elsevier Ltd. This is an open access article under the CC BY license (http://creativecommons.org/licenses/by/4.0/).

## Introduction

**Figure 1:**
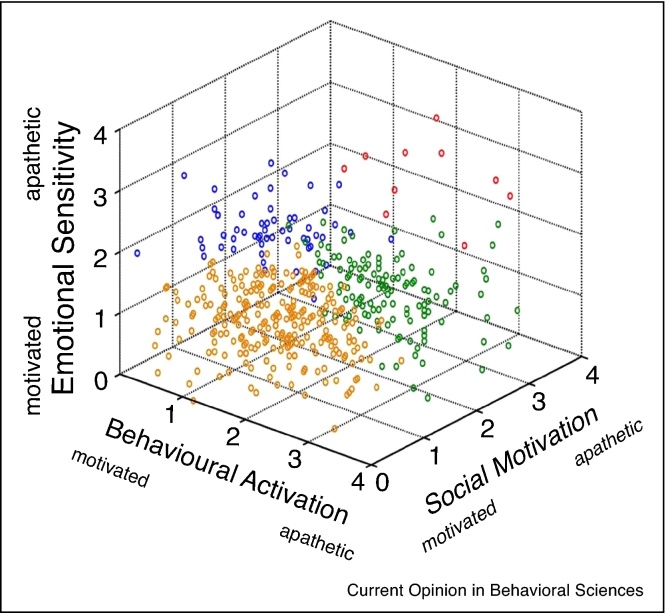
Distribution of multidimensional scores on the Apathy Motivation Index. 3D scatterplot of each healthy volunteer's mean rating on the multidimensional Apathy Motivation Index (AMI). Scores range from 0 to 4, with a higher mean score indicating greater apathy, for each of the three subscales: Behavioural Activation, Social Motivation and Emotional Sensitivity. Four subtypes of apathy-motivation along the scales were labelled motivated (orange), behaviourally/socially apathetic (green), emotionally apathetic (blue), and generally apathetic (red).

The most common neuropsychiatric symptom in patients with Alzheimer's disease (AD) is apathy [1•, 2], defined as loss of or diminished motivation in at least two out of three domains — *goal-directed behaviour*, *cognitive activity* or *emotion* — sufficient to cause significant impairment in everyday life [3]. Recent work in both healthy people and AD confirms the emerging view that apathy is not a single construct but a multidimensional disorder (Figure 1), that can also include distinct *social* as well as emotional deficits [4, 5].

With permission from [4].

The presence of apathy has been related to greater caregiver distress [6, 7, 8], decreased quality of life [9^•^], and increased morbidity [10]. Although distinguishable from each other, apathy often co-occurs with depression. While some symptoms of apathy and depression overlap (e.g. social withdrawal or reduced initiation), pure apathy is not associated with depressive symptoms such as guilt, hopelessness or sadness [11, 12, 13].

**Figure 2:**
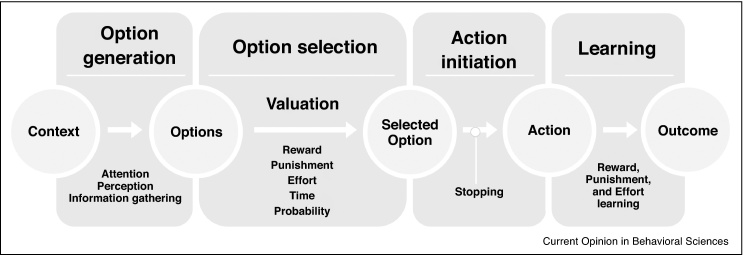
Schema of potential mechanisms involved in motivated behaviour and apathy. One scheme to dissect different behavioural components of apathy. Generation of behaviour might depend upon a number of components. First, people must be able to generate options for behaviour, with attentional and perceptual processes being important to produce possible behavioural options in a given context. Out of the options generated, one needs to be selected for action, based on values such as predicted reward, punishment, effort required, time involved, and probability of outcome associated with the option. Next that action needs to be initiated or, just as importantly, ongoing actions might need to be stopped, if they are less appropriate due to changes in the environment or context. They might also interfere with the initiation of action. Finally, if the action has been completed, the outcome of that behaviour needs to be compared with predictions made during valuation to modulate future choices.

Many of the conceptual and theoretical frameworks for understanding apathy — across brain disorders — have focused on goal-directed behaviour. Clinically, caregivers often observe that patients require prompting to do things, but left to their own devices they do not *self-initiate* behavioural activities. These types of observation have led to considerations of the underlying mechanisms that might be dysfunctional: ranging from a failure to generate options for behaviour, selecting between options depending upon valuation of their potential costs and benefits, action initiation and learning from outcomes (Figure 2). Analogous deconstruction of psychiatric syndromes has been put forward in the National Institute of Mental Health's Research Domain Criteria (Rod) initiative [14]. However, to date, as we shall see, very few behavioural mechanistic studies have been performed in AD.

With permission, adapted from [44].

## Prevalence and impact

A recent meta-analysis covering 25 studies reported a prevalence of apathy in AD ranging from 19% to 88%, with an overall mean prevalence of 49%. The significant heterogeneity across studies was shown to depend upon the apathy scale used, disease duration, Mini Mental State Examination (MMSE) score and education level [1^•^]. This might reflect difficulty to isolate apathy from symptoms of dementia, as well as symptoms of other neuropsychiatric conditions such as depression.

A number of both global and symptom-specific scales have been developed to assess the presence of neuropsychiatric symptoms, including uni-dimensional apathy measures such as the Neuropsychiatric Inventory (NPI) [15], the Apathy Evaluation Scale (AES) [16], the Dementia Apathy Interview and Rating Scale [17], and the Structured Clinical Interview for Apathy [13]. Multi-dimensional scales measuring cognitive, behavioural, affective or social aspects of apathy might be more useful for future studies aimed at assessing the prevalence of apathy in AD. Such multi-dimensional scales include the Apathy Inventory [18], the Dimensional Apathy Scale [5], the Lille Apathy Rating Scale (LARS) [19] and the Apathy Motivation Index [4].

Apathy appears to be associated with accelerated functional decline and increased morbidity in patients with AD. Caregiver-reported apathy, but not depression, predicts functional status in patients with AD, independent of age and cognitive function [20]. Moreover, clinician-rated apathy was related to more severe cognitive and functional decline, and reliably predicted time to death at any time point over the course of a 10-year longitudinal study [10]. Apathy may therefore be an important marker of disease progression in AD.

However, it is important to consider potential differences in the rating of the severity and impact of apathy between patients and caregivers. For example, in one longitudinal study over a 5-year follow-up period, an increase in apathy symptoms in patients with mild AD was not related to a change in *patient-reported* quality of life [9^•^]. In contrast, caregiver-reported patient quality of life decreased significantly. Thus, assessment of quality of life should ideally be corroborated with caregiver-rated questionnaires.

One recent study investigated which patient characteristics affected caregiver burden the most. Using a heterogeneous sample of memory clinic outpatients, the authors reported a strong effect of the severity of cognitive impairment and apathy on caregiver burden [6]. This was replicated in a recent study [8], although a previous study did not find this relationship [21]. A higher rating of the frequency and severity of apathy symptoms in AD patients by highly burdened caregivers was mediated by the use of disengagement coping strategies, suggesting that such strategies might render managing the apathetic patient more challenging for the caregiver [22].

## Behavioural studies

The few studies that have investigated the behavioural profile of apathy in AD provide evidence for apathy-associated impairments in reward-based decision-making and executive function, as well as altered behaviour on a task that implicitly measured social interest.

In a study on how apathy in AD is associated with decision-making, measured by performance on the Iowa Gambling Task (IGT), higher self-reported ratings on the action initiation dimension of the LARS were related to a disadvantageous decision-making profile [23]. In their analysis, the authors did not distinguish between groups of amnestic mild cognitive impairment, AD patients and healthy controls as there was no group-by-decision-making-profile interaction. Thus, there might be a global effect of apathy on reward-based decision-making that is not necessarily specific to AD pathology.

Another investigation examined cognitive and psychological profiles in apathetic AD and apathetic Parkinson's disease related dementia (PDD) patients and reported no significant differences in executive function between these groups [24]. Overall, however, apathetic patients were impaired in semantic fluency, motor response inhibition and abstract thinking when compared to non-apathetic patients with dementia. This trans-diagnostic study provides further evidence for an apathy-related executive impairment perhaps mediated by prefrontal cortex. On the basis of the similarity between apathetic AD and apathetic PDD patients, the authors argued for a general behavioural and cognitive dysexecutive syndrome that underlies apathy across neurodegenerative disorders.

Other investigators used a nonverbal visual scanning task to assess attentional bias in apathetic AD patients [25^•^]. Using eye-tracking, they showed that apathetic AD patients spent less time than their non-apathetic counterparts fixating on social, but not neutral, images. This might be considered in line with a social or emotional domain of apathy [4, 5]. However, in this investigation apathy was assessed with the uni-dimensional NPI, so no definitive conclusions can be drawn on the association with any subdomains of apathy.

## Neuroimaging studies

**Figure 3:**
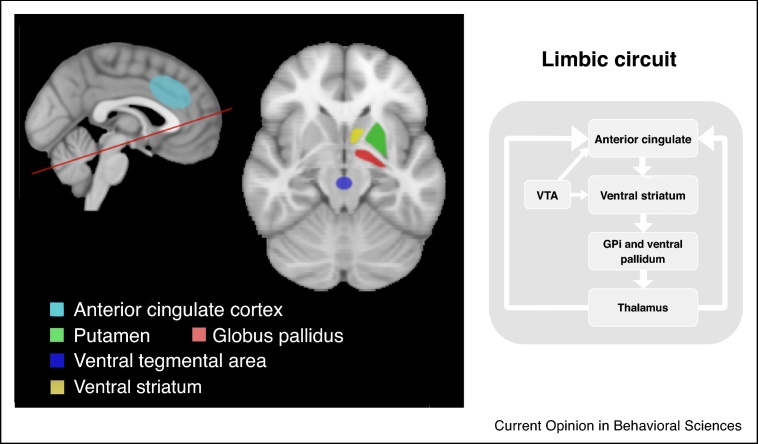
Schematic display of the frontostriatal circuit between anterior cingulate cortex and basal ganglia. The current model of basal ganglia function assumes a number of cortico-subcortico-cortico circuits connecting the basal ganglia with limbic, motor, oculomotor, and cognitive areas of the cortex. Shown here is the limbic circuit, consisting of connections from the ventral striatum to the thalamus, via the internal segment of the globus pallidus (GPi). The ventral striatum and cortex also receive dopaminergic inputs from the ventral tegmental area (VTA) via the mesolimbic pathway. GPi = globus pallidus interna. VTA = ventral tegmental area.

The behavioural studies discussed above suggest the involvement of prefrontal dysfunction, potentially associated with deficits within frontostriatal circuits (Figure 3). Indeed, several neuroimaging studies on apathy in AD, discussed in three recent comprehensive reviews, point to areas that are considered part of this circuit, including the anterior cingulate cortex (ACC), prefrontal cortex (PFC) and parts of the basal ganglia [26, 27, 28•]. The frontostriatal circuit, linking ventral striatum to dorsal ACC via the ventral pallidum and thalamus, may be crucially involved in effort-based decision making and executive functions. Disruption of this circuit has been hypothesised to play a pivotal role in apathy across neurodegenerative disorders [28^•^].

However, recent reports in AD have also implicated other regions and show how variable findings can be. In a cross-sectional sample of patients (*N* = 37) with either possible or probable AD, no association between apathy and grey matter atrophy was found [29]. As the mean age of participants in this study was >82 years and all had been recruited from a nursing home, generalised atrophy due to advanced age and dementia severity, as well as small sample size, might have influenced the result.

Similarly, a cross-sectional analysis from the Alzheimer's Disease Neuroimaging Initiative (ADNI) dataset (*N* = 188 AD; 395 MCI and 229 controls) found no significant relationship between regional atrophy and apathy in patients with mild cognitive impairment (MCI) and AD [30]. The longitudinal analysis revealed that progression of apathy over time was associated with reduced cortical thickness in bilateral inferior temporal cortex. As this finding had not been reported before, the authors suggest it may reflect an early-onset form of apathy in AD, since they did not distinguish between MCI and AD patients in their analyses.

Abnormal white matter integrity in AD patients with apathy but not depression, using tract-based spatial statistics (TBSS), has also been reported [31]. Compared with non-apathetic AD patients, those with apathy (*N* = 30 in each group) showed evidence of white matter changes in the corpus callosum, which was also correlated with severity of apathy. Severity of apathy was further correlated with loss of integrity of the left anterior and posterior cingulum, right superior longitudinal fasciculus, and bilateral uncinate fasciculus. The association between apathy and white matter damage in the corpus callosum was found in another study [29], which also reported an association between severity of apathy and damage in the internal capsule. Interpretation of these findings is not straightforward: how much is related to apathy *per se* versus general disease severity remains to be established.

One recent investigation used positron emission tomography with 18F fluorodeoxyglucose (FDG-PET) to examine how brain metabolism is associated with apathy in AD cases [32^•^]. The researchers reported a relationship between apathy and right ACC hypometabolism in AD. However, the patients in this sample (*N* = 42 AD cases) were in an advanced stage of dementia. Again, disease severity and generalised brain pathology unrelated to apathy might have influenced the finding.

Another FDG-PET study analysing a subsample of the ADNI dataset (*N* = 95 AD; 203 MCI and 104 controls) reported a cross-sectional and longitudinal association between posterior cingulate hypometabolism and higher apathy scores. There was no relation between apathy and hypometabolism in the inferior temporal lobe or ACC [33], as was reported in the previously mentioned studies [30, 32•]. This finding must be interpreted with caution, because the researchers did not differentiate between healthy controls, MCI, or mild AD in their analysis. Specific associations of apathy in health versus dementia cannot therefore be distinguished.

Brain amyloid-β, imaged by PET, has also been deployed in a small sample of AD patients (*N* = 28) to investigate if there are relationships with neuropsychiatric symptoms [34]. A positive correlation was found between severity of apathy, indexed by the NPI, and amyloid-β deposition in medial and orbitofrontal areas, insula, and right ACC. There were no significant differences between the apathetic and non-apathetic groups regarding cognitive function and disease duration, which provides some evidence that the relationship between apathy and amyloid deposition might not simply be mediated by severity of AD.

## Treatment

Standard pharmacological treatment of apathy in AD to date uses cholinesterase inhibitors (ChIs) such as donepezil, which increase the level and duration of action of acetylcholine. AD, and potentially apathy in AD, is associated with cholinergic dysfunction caused by disconnection of cholinergic pathways. ChIs improve overall cognitive function modestly in AD [35], and some studies have reported slight beneficial effects for neuropsychiatric symptoms in AD as well [36]. However, ChIs have not yet proven to be effective for the treatment of apathy in AD in the long term. Two reviews found either no [35], or only slight [37] evidence for a beneficial effect of ChIs compared to placebo, although treatment duration rarely exceeded 24 weeks.

The results of a clinical trial that compared combined treatment of donepezil and the cholinergic precursor choline alphoscerate (*N* = 56) versus donepezil alone (*N* = 57) has demonstrated significantly lower ratings of apathy after 1 and 2 years in the combined treatment arm compared to donepezil-only. The findings were unrelated to global cognitive functioning, but were associated with executive function at baseline such that those with apathy and more intact executive function appeared to benefit most from the combination treatment [38^•^].

Some investigators have examined whether apathy in AD might be related to impaired dopaminergic neurotransmission [39]. They tested the benefits of methylphenidate, a drug that increases catecholamines (including dopamine) in the brain and reported a significant improvement of apathy symptoms after a treatment of 6 weeks, with modest side effects. A recent double-blind, randomised placebo-controlled trial for methylphenidate reproduced this improvement after 4, 8, and 12 weeks of treatment, with no differences in adverse effects between treatment and placebo groups [40]. However, others have reported drop-out due to more severe methylphenidate-related side effects [41].

A comprehensive meta-analysis of the reported pharmacological studies [42] provides little evidence for their effectiveness in treating apathy in AD, although it did not have access to the most recent methylphenidate data. The findings are likely to have been affected by the small number of studies, and heterogeneity in drugs, sample and apathy assessment. However, of the available medication, ChIs seem to be the most effective. Whether additional use of choline precursors or methylphenidate will prove to enhance these effects remains to be established.

In a non-pharmacological treatment study, the potential of long-term exercise in improving neuropsychiatric symptoms in AD has been assessed [43]. There was no significant effect of a 12 month, 120 min per week exercise programme on any of the symptoms measured by the NPI, including apathy. However, mean NPI scores were low, and it is not clear whether some of the apathetic AD patients might also have suffered from depression. Thus, a small effect and heterogeneous sample might have affected the results.

A recent review on non-pharmacological treatment of apathy in AD concluded that some improvements might be possible with one-to-one activities, kit-based activity interventions, matched-to-interest or individualised activity programmes, individualised cognitive rehabilitation, multisensory behaviour or music therapy, cognitive stimulation therapy, and art therapy [41]. Combined pharmacological and individualised non-pharmacological training might be an important avenue to explore in future treatment trials for apathy in AD.

## Conclusions

In this review, we have evaluated the most recent studies on apathy in AD, focusing on prevalence, impact on quality of life, behavioural and neuroimaging studies, and treatment options. Despite high, but variable prevalence [1^•^], and the evident relation to worse outcome [7, 9•, 10], we have a poor understanding of underlying cognitive and behavioural mechanisms. In the future, use of conceptual frameworks such as the one shown in Figure 2 for self-generation of goal-directed behaviour might prove to be useful in better phenotyping of patients. Studies that have investigated the behavioural and neuroanatomical profile of apathy in AD point to a role of frontostriatal circuits, specifically involving the ACC [28•, 29, 31, 34]. This is plausible considering the implication of these regions in cost-benefit decision making in both patients and healthy controls [28^•^].

Apathy in AD patients is most commonly treated with ChIs, but personalised non-pharmacological treatments have provided some promising results as well [41]. There is missing consensus between many of the reported studies, which may be due to several reasons. Apathy is often measured only as a secondary outcome by the NPI. In addition, only few investigations have made efforts to select patients with apathy but without depression. Finally, many neuroimaging studies have used small sample sizes and not controlled for general levels of disease severity. While apathy is increasingly recognised as the commonest neuropsychiatric symptom in AD, the characteristic behavioural and neuroanatomical profile of the apathy syndrome in AD has yet to be properly established. This might be crucial for development of better therapeutic interventions.

## Conflicts of interest

Nothing declared.

## References and recommended reading

Papers of particular interest, published within the period of review, have been highlighted as:

• of special interest

## References

[1•] Zhao Q.F., Tan L., Wang H.F., Jiang T., Tan M.S., Tan L., Xu W., Li J.Q., Wang J., Lai T.J. (2016). The prevalence of neuropsychiatric symptoms in Alzheimer's disease: systematic review and meta-analysis. J Affect Disord.

[2] Siafarikas N., Selbaek G., Fladby T., Šaltyt Benth J., Auning E., Aarsland D. (2017). Frequency and subgroups of neuropsychiatric symptoms in mild cognitive impairment and different stages of dementia in Alzheimer's disease. Int Psychogeriatrics.

[3] Robert P.H., Mulin E., Mallea P., David R. (2010). Apathy diagnosis, assessment, and treatment in Alzheimer's disease. CNS Neurosci Ther.

[4] Ang Y.-S., Lockwood P., Apps M.A.J., Muhammed K., Husain M. (2017). Distinct subtypes of apathy revealed by the Apathy Motivation Index. PLOS ONE.

[5] Radakovic R., Starr J.M., Abrahams S. (2017). A novel assessment and profiling of multidimensional apathy in Alzheimer's disease. J Alzheimer's Dis.

[6] Dauphinot V., Delphin-Combe F., Mouchoux C., Dorey A., Bathsavanis A., Makaroff Z., Rouch I., Krolak-Salmon P. (2015). Risk factors of caregiver burden among patients with Alzheimer's disease or related disorders: a cross-sectional study. J Alzheimer's Dis.

[7] Riedijk S.R., De Vugt M.E., Duivenvoorden H.J., Niermeijer M.F., Van Swieten J.C., Verhey F.R.J., Tibben A. (2006). Caregiver burden, health-related quality of life and coping in dementia caregivers: a comparison of frontotemporal dementia and Alzheimer's disease. Dement Geriatr Cogn Disord.

[8] Chen C.T., Chang C.-C., Chang W.-N., Tsai N.-W., Huang C.-C., Chang Y.-T., Wang H.-C., Kung C.-T., Su Y.-J., Lin W.-C. (2017). Neuropsychiatric symptoms in Alzheimer's disease: associations with caregiver burden and treatment outcomes. QJM Int J Med.

[9•] Hongisto K., Hallikainen I., Selander T., Törmälehto S., Väätäinen S., Martikainen J., Valimaki T., Hartikainen S., Suhonen J., Koivisto A.M. (2017). Quality of life in relation to neuropsychiatric symptoms in Alzheimer's disease: 5-year prospective ALSOVA cohort study. Int J Geriatr Psychiatry.

[10] Spalletta G., Long J.D., Robinson R.G., Trequattrini A., Pizzoli S., Caltagirone C., Orfei M.D. (2015). Longitudinal neuropsychiatric predictors of death in Alzheimer's disease. J Alzheimers Dis.

[11] Benoit M., Berrut G., Doussaint J., Bakchine S., Bonin-Guillaume S., Fremont P., Gallarda T., Krolak-Salmon P., Marquet T., Mekies C. (2012). Apathy and depression in mild Alzheimer's disease: a cross-sectional study using diagnostic criteria. J Alzheimers Dis.

[12] Benoit M., Andrieu S., Lechowski L., Gillette-Guyonnet S., Robert P.H., Vellas B., REAL-FR group (2008). Apathy and depression in Alzheimer's disease are associated with functional deficit and psychotropic prescription. Int J Geriatr Psychiatry.

[13] Starkstein S.E., Ingram L., Garau M.L., Mizrahi R. (2005). On the overlap between apathy and depression in dementia. J Neurol Neurosurg Psychiatry.

[14] Kozak M.J., Cuthbert B.N. (2016). The NIMH Research Domain Criteria Initiative: background, issues, and pragmatics. Psychophysiology.

[15] Cummings J.L. (1997). The Neuropsychiatric Inventory: assessing psychopathology in dementia patients. Neurology.

[16] Marin R.S., Biedrzycki R.C., Firinciogullari S. (1991). Reliability and validity of the apathy evaluation scale. Psychiatry Res.

[17] Strauss M.E., Sperry S.D. (2002). An informant-based assessment of apathy in Alzheimer disease. Neuropsychiatry Neuropsychol Behav Neurol.

[18] Robert P.H., Clairet S., Benoit M., Koutaich J., Bertogliati C., Tible O., Caci H., Borg M., Brocker P., Bedoucha P. (2002). The Apathy Inventory: assessment of apathy and awareness in Alzheimer's disease, Parkinson's disease and mild cognitive impairment. Int J Geriatr Psychiatry.

[19] Sockeel P., Dujardin K., Devos D., Deneve C., Destee A., Defebvre L. (2006). The Lille apathy rating scale (LARS), a new instrument for detecting and quantifying apathy: validation in Parkinson's disease. J Neurol Neurosurg Psychiatry.

[20] You S.C., Walsh C.M., Chiodo L.A., Ketelle R., Miller B.L., Kramer J.H. (2015). Neuropsychiatric symptoms predict functional status in Alzheimer's disease. J Alzheimers Dis.

[21] Allegri R.F., Sarasola D., Serrano C.M., Taragano F.E., Arizaga R.L., Butman J., Loñ L. (2006). Neuropsychiatric symptoms as a predictor of caregiver burden in Alzheimer's disease. Neuropsychiatr Dis Treat.

[22] Garcia-Alberca J.M., Lara J.P., Garrido V., Gris E., Gonzalez-Herero V., Lara A. (2014). Neuropsychiatric symptoms in patients with Alzheimer's disease: the role of caregiver burden and coping strategies. Am J Alzheimers Dis Other Dementias.

[23] Bayard S., Jacus J.P., Raffard S., Gely-Nargeot M.C. (2014). Apathy and emotion-based decision-making in amnesic mild cognitive impairment and Alzheimer's disease. Behav Neurol.

[24] Grossi D., Santangelo G., Barbarulo A.M., Vitale C., Castaldo G., Proto M.G., Siano P., Barone P., Trojano L. (2013). Apathy and related executive syndromes in dementia associated with Parkinson's disease and in Alzheimer's disease. Behav Neurol.

[25•] Chau S.A., Chung J.T., Herrmann N., Eizenman M., Lanctot K.L. (2016). Apathy and attentional biases in Alzheimer's disease. J Alzheimers Dis.

[26] Stella F., Radanovic M., Aprahamian I., Canineu P.R., de Andrade L.P., Forlenza O. (2014). Neurobiological correlates of apathy in Alzheimer's disease and mild cognitive impairment: a critical review. J Alzheimers Dis.

[27] Theleritis C., Politis A., Siarkos K., Lyketsos C.G. (2014). A review of neuroimaging findings of apathy in Alzheimer's disease. Int Psychogeriatrics.

[28•] Le Heron C., Apps M.A.J., Husain M. (2017). The anatomy of apathy: a neurocognitive framework for amotivated behavior. Neuropsychologia.

[29] Aguera-Ortiz L., Hernandez-Tamames J.A., Martinez-Martin P., Cruz-Orduna I., Pajares G., Lopez-Alvarez J., Osorio R.S., Sanz M., Olazaran J. (2016). Structural correlates of apathy in Alzheimer's disease: a multimodal MRI study. Int J Geriatr Psychiatry.

[30] Donovan N.J., Wadsworth L.P., Lorius N., Locascio J.J., Rentz D.M., Johnson K.A., Sperling R.A., Marshall G.A. (2014). Regional cortical thinning predicts worsening of apathy and hallucinations across the Alzheimer's disease spectrum. Am J Geriatr Psychiatry.

[31] Hahn C., Lee C. (2013). Apathy and white matter integrity in Alzheimer's disease: a whole brain analysis with tract-based spatial statistics. Int Psychogeriatrics.

[32•] Fernandez-Matarrubia M., Matias-Guiu J.A., Cabrera-Martin M.N., Moreno-Ramos T., Valles-Salgado M., Carreras J.L., Matias-Guiu J. (2017). Different apathy clinical profile and neural correlates in behavioral variant frontotemporal dementia and Alzheimer's disease. Int J Geriatr Psychiatry.

[33] Gatchel J.R., Donovan N.J., Locascio J.J., Becker J.A., Rentz D.M., Sperling R.A., Johnson K.A., Marshall G.A., ADNI (2017). Regional 18F-fluorodeoxyglucose hypometabolism is associated with higher apathy scores over time in early Alzheimer disease for the Alzheimer's Disease Neuroimaging Initiative. Am J Geriatr Psychiatry.

[34] Mori T., Shimada H., Shinotoh H., Hirano S., Eguchi Y., Yamada M., Fukuhara R., Tanimukai S., Zhang M., Kuwabara S. (2014). Apathy correlates with prefrontal amyloid ß deposition in Alzheimer's disease. J Neurol Neurosurg Psychiatry.

[35] Kobayashi H., Ohnishi T., Nakagawa R., Yoshizawa K. (2016). The comparative efficacy and safety of cholinesterase inhibitors in patients with mild-to-moderate Alzheimer's disease: a Bayesian network meta-analysis. Int J Geriatr Psychiatry.

[36] Wang J., Yu J.T., Wang H.F., Meng X.F., Wang C., Tan C.C., Tan L. (2015). Pharmacological treatment of neuropsychiatric symptoms in Alzheimer's disease: a systematic review and meta-analysis. J Neurol Neurosurg Psychiatry.

[37] Rea R., Carotenuto A., Fasanaro A.M., Traini E., Amenta F. (2014). Apathy in Alzheimer's disease: any effective treatment?. Sci World J.

[38•] Rea R., Carotenuto A., Traini E., Fasanaro A.M., Manzo V., Amenta F. (2015). Apathy treatment in Alzheimer's disease: interim results of the ASCOMALVA trial. J Alzheimers Dis.

[39] Rosenberg P.B., Lanctot K.L., Drye L.T., Herrmann N., Scherer R.W., Bachman D.L., Mintzer J.E., The ADMET Investigators (2013). Safety and efficacy of methylphenidate for apathy in Alzheimer's disease: a randomized, placebo-controlled trial. J Clin Psychiatry.

[40] Padala P.R., Padala K.P., Lensing S.Y., Ramirez D., Monga V., Bopp M.M., Roberson P.K., Dennis R.A., Petty F., Sullivan D.H. (2017). Methylphenidate for apathy in community-dwelling older veterans with mild Alzheimer's disease: a double-blind, randomized, placebo-controlled trial. Am J Psychiatry.

[41] Theleritis C., Siarkos K., Katirtzoglou E., Politis A. (2017). Pharmacological and nonpharmacological treatment for apathy in Alzheimer disease: a systematic review across modalities. J Geriatr Psychiatry Neurol.

[42] Sepehry A.A., Sarai M., Hsiung G.Y.R. (2017). Pharmacological therapy for apathy in Alzheimer's disease: a systematic review and meta-analysis. Can J Neurol Sci.

[43] Ohmana H., Savikko N.R.N., Strandberg T.E., Kautiainen H., Raivio M.M., Laakkonen M.L., Tilvis R., Pitkala K.H. (2017). Effects of frequent and long-term exercise on neuropsychiatric symptoms in patients with Alzheimer's disease — secondary analyses of a randomized, controlled trial (FINALEX). Eur Geriatr Med.

[44] Sinha N., Manohar S., Husain M. (2013). Impulsivity and apathy in Parkinson's disease. J Neuropsychol.

